# Pictograms to aid laypeople in identifying the addictiveness of gambling products (PictoGRRed study)

**DOI:** 10.1038/s41598-022-26963-9

**Published:** 2022-12-29

**Authors:** Amandine Luquiens, Morgane Guillou, Julie Giustiniani, Servane Barrault, Julie Caillon, Helena Delmas, Sophia Achab, Bruno Bento, Joël Billieux, Damien Brevers, Aymeric Brody, Paul Brunault, Gaëlle Challet-Bouju, Mariano Chóliz, Luke Clark, Aurélien Cornil, Jean-Michel Costes, Gaetan Devos, Rosa Díaz, Ana Estevez, Giacomo Grassi, Anders Hakansson, Yasser Khazaal, Daniel L. King, Francisco Labrador, Hibai Lopez-Gonzalez, Philip Newall, José C. Perales, Aurélien Ribadier, Guillaume Sescousse, Stephen Sharman, Pierre Taquet, Isabelle Varescon, Cora Von Hammerstein, Thierry Bonjour, Lucia Romo, Marie Grall-Bronnec

**Affiliations:** 1 Department of Addictology, CHU Nîmes, Univ Montpellier, Nîmes, France; 2 CESP, Univ. Paris-Sud, UVSQ, INSERM, Université Paris-Saclay, Villejuif, France; 3 EA 7479 SPURBO, CHRU BREST, Université de Bretagne Occidentale, Brest and Addictologie, Brest, France; 4 CHU de Besançon, Besançon, France; 5 QualiPsy, EE 1901, Université de Tours, Tours, France; 6 Service d’Addictologie Universitaire, CSAPA-37, CHRU de Tours, Tours, France; 7 Department of Addictology and Psychiatry Nantes, Inserm U1246, CHU Nantes, Université de Nantes, Université de Tours, Nantes, France; 8 Pôle Addiction et Précarité, Centre Hospitalier Guillaume Régnier, Rennes, France; 9 WHO Collaborating Centre for Treatment and Research in Mental Health, University of Geneva, Geneva, Switzerland; 10 IAJ - Instituto de Apoio ao Jogador, Lda, Portugal; 11 Institute of Psychology, University of Lausanne, Lausanne, Switzerland; 12 Addiction Medicine, Centre for Excessive Gambling, Lausanne University Hospitals (CHUV), Lausanne, Switzerland; 13 Louvain Experimental Psychopathology (LEP), Psychological Science Research Institute, Louvain-La-Neuve, Belgium; 14 EPITA, LRE MNSHS, Paris, France; 15 Service d’Addictologie Universitaire, Équipe de Liaison et de Soins en Addictologie, CHRU de Tours, Tours, France; 16 UMR 1253, iBrain, Inserm, Université de Tours, Tours, France; 17 QualiPsy, EE, Université de Tours, 1901 Tours, France; 18 Gambling and Technological Addictions Research Unit, University of Valencia, Valencia, Spain; 19 Department of Psychology, Centre for Gambling Research at UBC, University of British Columbia, Vancouver, BC Canada; 20 Centre for Excessive Gambling, Université Catholique de Louvain, Lausanne University Hospitals (CHUV), Lausanne, Switzerland; 21 Observatoire des Jeux, Paris, France; 22 Grand Hôpital de Charleroi (GHdC), Charleroi, Belgium; 23 Psychological Science Research Institute, Université Catholique de Louvain, Louvain-la-Neuve, Belgium; 24 Scientific Research and Publication Cell (CRPS), Le Beau Vallon, Namur, Belgium; 25 Centre Hospitalier Le Domaine, ULB, Braine-L’Alleud, Belgium; 26 Service Universitaire d’Addictologie de Lyon (SUAL), CH Le Vinatier, 69500 Bron, France; 27 Child and Adolescent Psychiatry and Psychology Department, Hospital Clínic Universitari de Barcelona, Barcelona, Spain; 28 University of Deusto, Bilbao, Spain; 29 BRAIN CENTER FIRENZE, Florence, Italy; 30 Clinical Addiction Research Unit, Faculty of Medicine, Malmö Addiction Center, Lund University - Gambling Disorder Unit, Region Skåne, Sweden; 31 Addiction Medicine, Department of Psychiatry, Lausanne University Hospital and Lausanne University, Lausanne, Switzerland; 32 College of Education, Psychology, & Social Work, Flinders University, Adelaide, Australia; 33 Faculty of Psychology, Complutense University, Madrid, Spain; 34 Faculty of Information and Communication, Universitat de Barcelona, Barcelona, Spain; 35 CQUniversity, Bundaberg, Australia; 36 Department of Experimental Psychology Mind, Brain and Behavior Research Centre (CIMCYC), University of Granada, Granada, Spain; 37 Département de Psychologie, EE 1901 - Equipe Qualipsy « Qualité de vie et Santé Psychologique », Université de Tours, Tours, France; 38 Lyon Neuroscience Research Center—INSERM U1028—CNRS UMR5292, PSYR2 Team, University Lyon 1, Lyon, France; 39 National Addiction Centre, King’s College, London, UK; 40 Psychiatry and Addiction Medicine Department, CHU Lille, 59000 Lille, France; 41 Univ. Lille, ULR, 4072 Lille, France; 42 PSITEC—Psychologie: Interactions Temps Émotions Cognition, 59000 Lille, France; 43 Laboratoire de Psychopathologie et Processus de Santé, Université de Paris, 92100 Boulogne Billancourt, France; 44 APEMAC, Équipe EPSAM, Université de Lorraine, 57000 Metz, France; 45 EA 4430 Clipsyd, University Paris Nanterre, Nanterre, France

**Keywords:** Human behaviour, Risk factors

## Abstract

The structural addictive characteristics of gambling products are important targets for prevention, but can be unintuitive to laypeople. In the PictoGRRed (Pictograms for Gambling Risk Reduction) study, we aimed to develop pictograms that illustrate the main addictive characteristics of gambling products and to assess their impact on identifying the addictiveness of gambling products by laypeople. We conducted a three-step study: (1) use of a Delphi consensus method among 56 experts from 13 countries to reach a consensus on the 10 structural addictive characteristics of gambling products to be illustrated by pictograms and their associated definitions, (2) development of 10 pictograms and their definitions, and (3) study in the general population to assess the impact of exposure to the pictograms and their definitions (*n* = 900). French-speaking experts from the panel assessed the addictiveness of gambling products (*n* = 25), in which the mean of expert’s ratings was considered as the true value. Participants were randomly provided with the pictograms and their definitions, or with a standard slogan, or with neither (control group). We considered the control group as representing the baseline ability of laypeople to assess the addictiveness of gambling products. Each group and the French-speaking experts rated the addictiveness of 14 gambling products. The judgment criterion was the intraclass coefficients (ICCs) between the mean ratings of each group and the experts, reflecting the level of agreement between each group and the experts. Exposure to the pictograms and their definition doubled the ability of laypeople to assess the addictiveness of gambling products compared with that of the group that read a slogan or the control group (ICC = 0.28 vs. 0.14 (Slogan) and 0.14 (Control)). Laypeople have limited awareness of the addictive characteristics of gambling products. The pictograms developed herein represent an innovative tool for universally empowering prevention and for selective prevention.

## Introduction

Addictive disorders represent a major public health challenge with considerable human and social costs. Gambling disorders cause direct individual damage to mental and physical health, with financial, socio-professional, collective, cultural, and legal consequences^1^. The transition between recreational and problematic gambling is multifactorial^2^. Many studies have focused on individual or contextual vulnerability factors^3–5^, yet the structural components of gambling products also have a direct influence on their riskiness and addictive potential, as demonstrated in neuroscience experiments^6,7^. Our study conceptualizes gambling disorder as defined in the chapter on disorders due to substance use or addictive behaviors of the 11th edition of the *International Statistical Classification of Diseases and Related Health Problems* (ICD-11). Thus, addictive components constitute any that could contribute to the three dimensions of gambling disorder: “1. impaired control over gambling; 2. increasing priority given to gambling; 3. continuation or escalation of gambling despite the occurrence of negative consequences”^8^. Parke et al. performed a literature review and identified several components that contribute to addictiveness of gambling products: game characteristics, ambient characteristics, speed and frequency of gambling opportunities, reward characteristics, cost characteristics, payment and accounting characteristics, and information characteristics^6^. Moreover, advertisements of gambling products modify consumers’ behavior to normalize gambling products as ordinary commodities^9^. Messages promoting ease of gambling, including those regarding bonuses and rapid cash out, are thought to be particularly risky. Some tools have been developed to assess the global level of risk of a particular gambling product. The ASTERIG tool generates weighted scores on the addictive risk of gambling products according to 10 components: event frequency, interval of payback, jackpot, continuity of playing, chance of winning a profit, availability, multiple playing/stake opportunities, variable stake amount, sensory product design, and near wins^10^. This tool has been validated and improved by international experts^11^. The ASTERIG tool has the benefit of indicating which components increase the risk of a particular gambling product. Nevertheless, these concepts are complex, and ASTERIG is targeted toward medical and psychological scientists, lawyers, judges, and policymakers rather than the general population.

Misconceptions about gambling are priority targets for prevention, but can be unintuitive and hard to explain^12^. Existing universal and selective prevention programs provide low-cost tools to target at-risk individuals who may not yet have engaged in problem gambling, particularly adolescents. Prevention programs on problem gambling that target the general population include components on improving knowledge about gambling and problem gambling^13,14^. Prevention activities like slogans have been widely used by authorities and public health institutions to prevent gambling disorder, yet their efficacy depends on the wording and the targeted population^15^. All prevention slogans are not efficient in se, and can lead to unexpected effects among gamblers^16^. However, there is a need for an independent tool to inform and empower the general population about the potential risks of gambling products. Such a tool ought to be broadly available to, and understandable by, laypeople, including those who are not yet engaged in gambling behaviors. Pictograms have been used effectively as prevention tools in health care^17,18^, as they are easily understandable and convey simple messages. Pictograms can help in decision making by offering cues to users, particularly to signal dangerousness^19^. For example, in the video gaming field, the PEGI pictograms target the general population, in particular parents, in order to identify the characteristics of the risk components of video games (e.g., violence, sexual content)^20^. However, interpretation of potentially contradictory information between pictograms and other sources of information, such as advertisements, varies between individuals and affects decision making^21^.

The primary objective of this project was to develop pictograms and their associated definitions that would be understandable by laypeople to illustrate the addictive components of gambling products and of messages linked to gambling products, i.e., advertisements and instructions. The secondary objective was to assess the impact of exposure to these pictograms and their definitions on the general population’s assessment of addictiveness of gambling products. Our hypothesis was that exposure to the pictograms and their definitions has the potential to increase the ability of laypeople to estimate the addictiveness of gambling products, i.e., to reconcile their estimation with that of the experts, as compared with no exposure or with exposure to a standard prevention slogan.

## Methods

The PictoGRRed (Pictograms for Gambling Risk Reduction) study had three steps: (1) use of the Delphi method, (2) development of pictograms, and (3) validation in the general population of the impact of exposure to the pictograms and their definitions compared with two conditions: a standard warning slogan or nothing (control group).

### Delphi consensus method

We used an online Delphi process and structured consensus method that involved experts in gambling disorder, to reach a consensus on the 10 structural addictive characteristics of gambling products to be illustrated by pictograms and their associated definitions. The Delphi method is a well-established method to reach a consensus between experts, widely used in mental health^22,23^. A Delphi panel appeared to be the optimal method, given the diversity of addictive gambling characteristics, the gap between fundamental research and practical behavior in ecological environments, and the lack of published evidence on a limited set of characteristics and of evidence on addictive gambling characteristics that would be critical for empowerment of laypeople.

### Constitution of the panel of experts

First, a steering committee was formed, consisting of eight members of the RNPSJP (French network for gambling disorder health care and prevention; co-authors AL, MG, SB, JC, HD, JG, LR, MGB). The steering committee identified experts for the expert panel, comprising international experts in gambling disorder, including clinicians, researchers, regulation authorities, and members of Gamblers Anonymous. Experts were eligible to join the panel if they (1) were a professional specialized in gambling disorder (health professional, researcher, or regulator) or member of a self-help group of problem gamblers, (2) were not employed by the gambling industry, (3) agreed to being listed in the publication reporting this project, (4) agreed to complete a declaration of interest, and (5) were willing to participate in both the item elicitation and the Delphi process. The steering committee contacted all members of the French network for gambling disorder health care and prevention. This association covers all French clinicians specialized in gambling disorder who work in referral centers with dedicated public funding for gambling disorder health care. Second, an initial list of 32 international experts was elicited by the French specialists and collected by the steering committee, including two employees of the French gambling regulation and in charge of responsible gambling, two active members of the French association “Gamblers anonymes,” affiliated with Gamblers Anonymous, and 28 clinicians and/or researchers with at least one publication on gambling. To ensure diversity of the panel, we chose experts from a range of fields, including psychiatrists, neuropsychologists, psychologists, sociologists, epidemiologists, and research engineers. Third, the snowball method was used. The listed experts were invited by email to participate and to propose other health professionals and researchers specialized in gambling disorder (i.e. health professionals working in a unit specialized in gambling disorder, or a researcher with at least one publication in English in a peer reviewed journal on gambling disorder) whom they could endorse and who met the 5 criteria described earlier. The list was then extended to 66 experts. Ten of the solicited experts refused or did not answer; therefore, 56 experts from 13 different countries and 3 continents were identified and agreed to participate. Represented countries were: Australia, Canada, Switzerland, France, Spain, Portugal, United Kingdom, Luxembourg, Belgium, Netherlands, Italy, Sweden, Germany. Participants were informed that their responses during the Delphi phase would be anonymized and that only the responses of the whole group would be reported and communicable.

### Online Delphi consensus method

The online Delphi method allowed experts to reach consensus through a structured and iterative process (rounds) by contributing and commenting electronically (using REDCap^®^), between June 10, 2020 and July 27, 2020. In each round, all experts could view a summary of the group opinion, allowing them to modify their responses in the following rounds until they reached a consensus. We aimed to identify 10 priority structural addictive characteristics of gambling products and of messages linked to gambling products, to be illustrated by pictograms for psycho-education in the general population. We chose a maximum target of 10 pictograms for clarity of use and recall for the general population, following the number chosen for the PEGI descriptors^20^. An initial list of 33 structural characteristics and their definitions was extracted from a literature review on structural addictive characteristics of gambling products^6^. The initial list could be extended by the experts (Table 1).

**Table 1:** Final 10 pictograms and their reworded definitions for laypeople.

Addictive structural characteristics of gambling products and related messages	Pictogram	Definition reworded for laypeople: “This pictogram regards a gambling game.…”
High event frequency (virtually unlimited opportunity to play)		“… where it is possible to bet several times in a row so that the gambler can continue to play practically without a break.”
Fast game (high event frequency and limited scope for decision making)		“…where it is possible to wager frequently but with a limited time to think before each wager.”
In-game actions overly suggesting control of the outcome (in-game actions suggesting control of chance, whereas they do not or only poorly influence the outcome)		“…with actions that give the player the feeling that he or she can control the outcome (win/loss) when this is not the case.”
In-running betting (e.g., live betting)		“…where the gambler plays at the same time as the action is in progress (e.g., live betting, sports betting during a match).”
Short payout interval (includes rapid provision of money and automatically adding money win to credit)		“…with a short payout period: the money won is very quickly paid into the gambler's account, inviting the gambler to keep on gambling.”
Losses disguised as wins (gain inferior to the amount staked)		“…where losses are disguised as gains. For example, you buy a gambling game for 3 euros. You scratch and the gambling game indicates that you have won 1 euro, when in reality you have lost 2 euro.”
Near miss and equivalent of near miss situations (outcome that suggests an outcome close to a winning situation, whereas outcome is binary: win or lose)		“…in which the outcome suggests to the gambler that he/she has ‘almost’ won, while the outcome is binary (win or lose) and he/she has lost.”
Messages associated with the game suggesting control of chance (advertisement, instruction) (e.g., advertisement claiming that you have particular ability over the others to win)		“…in which the advertising messages or instructions suggest that the gambler has control over chance (e.g., the advertisement claims that the gambler has a greater ability to win than others).”
Unlimited temporal access to the game (24/7 venue, online…)		“… with an access unlimited in time (24/7, online).”
Messages promoting ease of gambling (including bonus and rapid cash-out messages)		“… for which advertising messages or instructions encourage gambling (e.g., ‘safe first bet,’ bonus, rapid cash-out).”

**Figure 1:**
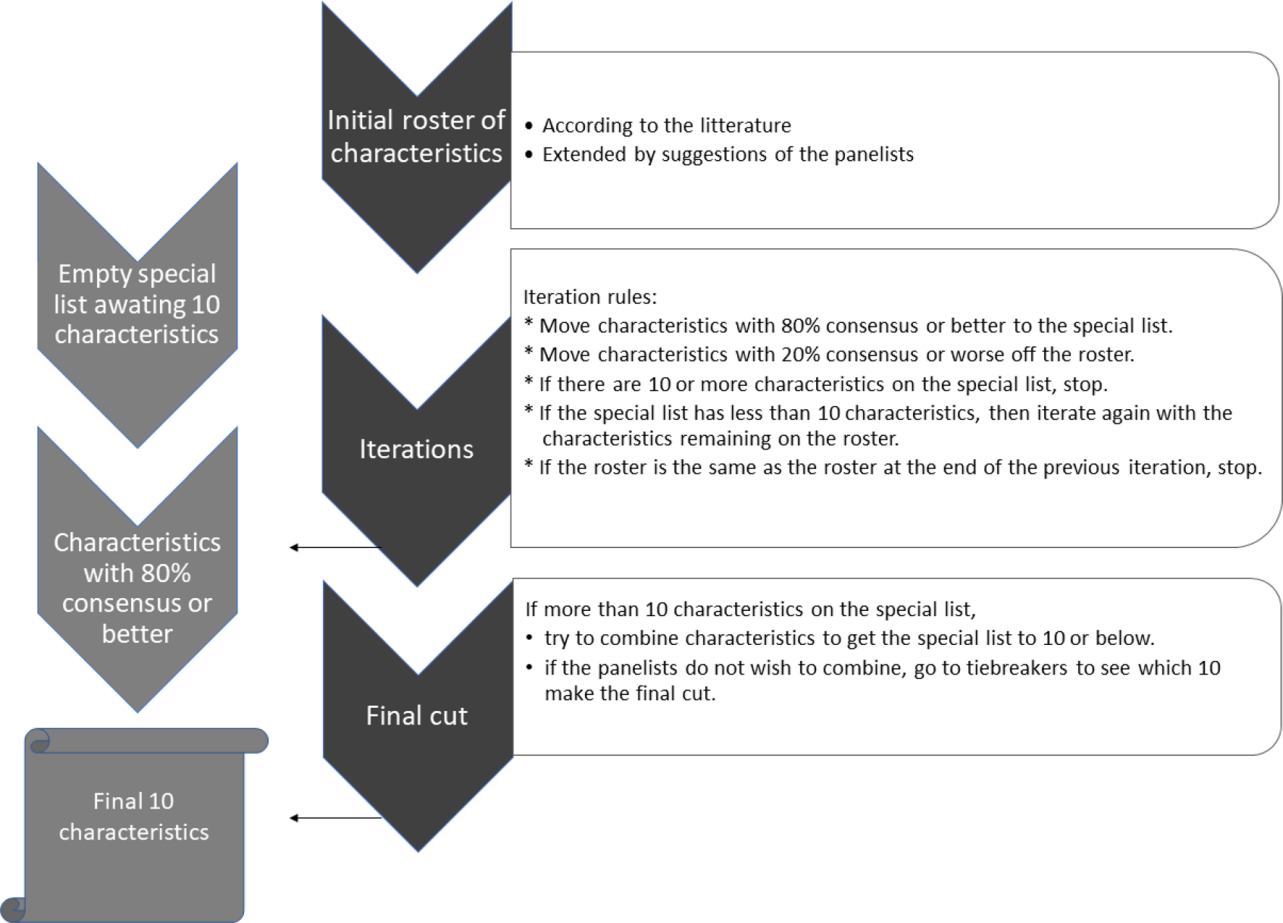
Delphi process and rules.

The Delphi process and its rules are presented in Fig. 1. In the Delphi method, multiple rounds are anticipated to occur to reach consensus. The instruction for each round was as follows:

Rating of addictive characteristics: For each of the following candidate characteristics, please indicate the level of priority, i.e. the most important and appropriate concepts to be illustrated with pictograms to be used as psycho-educational tools in the general population. Please note that some characteristics overlap or include others. The objective here is to reach a consensus on up to 10 priority characteristics.

In each round, the experts rated the priority of each structural characteristic on a 5-point Likert scale (not a priority, low priority, medium priority, high priority, essential; Ref.^23^.

### Development of the pictograms and their definitions

A graphic artist, who was also qualified as a medical doctor specialist in addiction science (TB), designed pictograms to represent each of the 10 structural characteristics retained from the Delphi procedure. The steering committee discussed the proposed pictograms in order to choose 10 with satisfying face and content validity. Wordings of definitions were revised by 10 non-specialists in addiction from a firm that specializes in surveys of laypeople (Kantar Public) in order to avoid literacy errors and to optimize legibility.

### Test of the impact of the pictograms and their definitions in the general population

#### Design and population

We conducted an online survey in a sample of 900 laypeople from the French general population over 18 years old. The sample was randomly selected from a large probabilistic panel in Europe that provides robust, representative survey data not limited to individuals who are actively seeking online surveys to complete, thus limiting the risk of sample bias. There were no exclusion criteria. Participants were compensated for their time with a gift card after completion.

#### Design

Participants were randomly assigned to three groups stratified by age, sex, socio-professional category of the main breadwinner, and Problem Gambling Severity Index (PGSI) score (< 5 or ≥ 5; Refs.^24–26^: 2 groups exposed to a prevention activity, i.e. the Pictogram group and the Slogan group, and a Control group. Participants in the Pictogram group viewed the 10 pictograms and their definitions in random order with the following instruction:

Each pictogram represents an addictive component of gambling products. These components may or may not be present in a particular gambling product, and their presence increases the risk of addiction to that gambling product. Please take the time to carefully look at each pictogram and read the text associated with the pictogram.

In the Slogan group, participants were shown the following current responsible gambling slogan: “Gambling involves risks: debt, isolation, addiction. For help, call 09741513133.” The Control group proceeded directly to the sociodemographic questions and was considered to represent the baseline knowledge of laypeople.

After exposure to the pictograms and their definition or to the slogan, or after no exposure, participants from each group were asked to rate whether their desire to gamble had changed. They were then asked to assess the addictiveness of 14 French current gambling products displayed in random order and illustrated with a photograph of the product. The photographs were presented without explanation or information other than the type of gambling game: offline/online lottery, offline/online speed lottery, scratching, horse race betting, sport betting, poker, and casino games. Participants could remember and assimilate the information previously delivered in the Pictogram, Slogan, or Control groups to assess the addictiveness of the gambling products themselves.

In parallel, French-speaking experts of the Delphi panel (*n* = 25) were also asked to assess the addictiveness of the same 14 gambling products, according to their expertise and personal judgement. We considered the experts’ opinion as the data with the highest level of evidence and the “true rating” for the analyses.

#### Data collection

Data collection included the following: age, sex, employment status, academic level, and place of residency. Gambling habits were also collected: last 12 months’ use of offline and online lottery, scratching, horse race betting, sport betting, poker, casino games, or none, as well as scores on Problem Gambling Severity Index of the Canadian Problem Gambling Index (PGSI).

Desire to gamble was measured as “decreased” (− 1), “no change” (0), or “increased” (+ 1). Addictiveness of each of the14 French current gambling products was assessed on a 5-point Likert scale from 0 (not addictive) to 4 (highly addictive) responding the question “After seeing these pictograms/slogan/[], how addictive do you think the following game is?”.

### Statistical analyses

**Figure 2:**
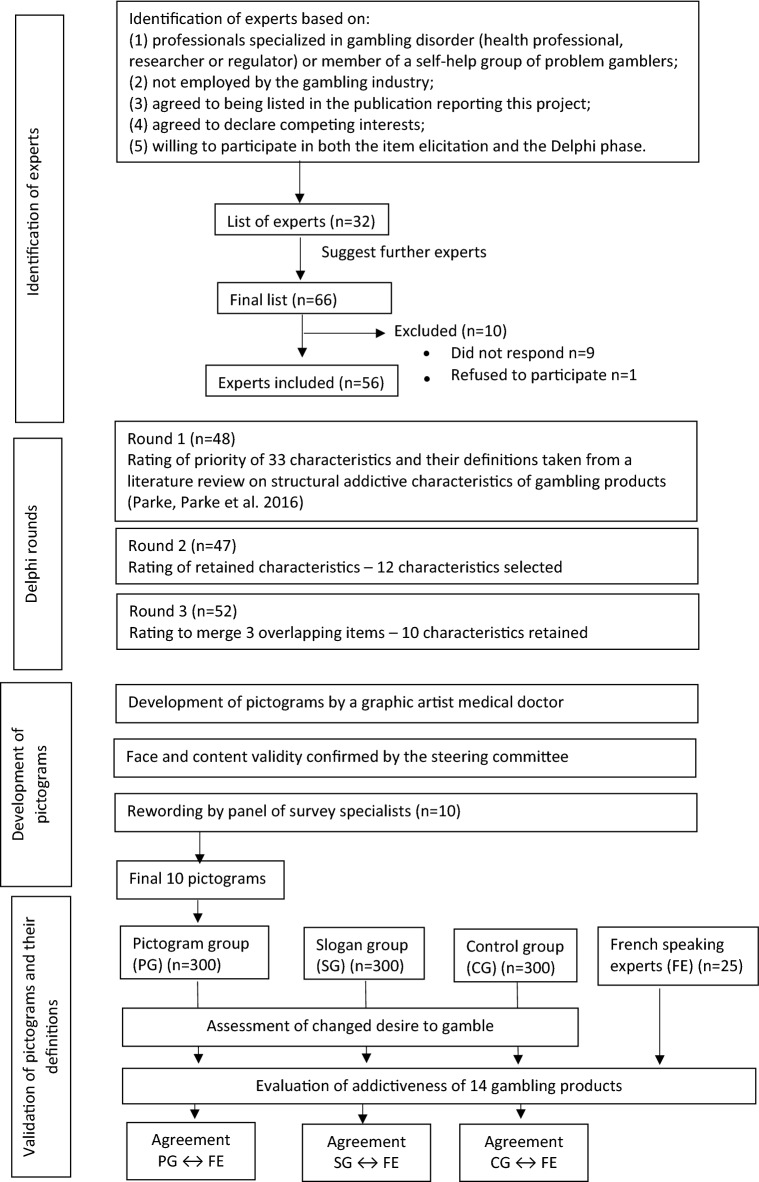
Flow chart.

Descriptive analyses were conducted for all variables by group. We used the following operational definition of agreement: reliable raters agree with the “true” rating of addictiveness, determined by the mean rating of the experts for each of the 14 gambling products. The three groups of laypeople were considered to be three raters, and mean addictiveness was calculated by group for the 14 gambling products. The primary outcome was therefore the 2–2 groups-experts inter-rater coefficient of addictiveness on the 14 gambling products (mean of each group for each of 14 products , as calculated by the intra-class coefficient (ICC); Two-Way Random-Effects Model, R package “irr”, “icc”, twoways, agreement, average; Refs.^27,28^. We hypothesized that PictoGRRed pictograms and their definitions would increase the agreement between laypeople and experts, where the experts’ opinion was considered the “true rating”; more precisely, the Pictogram group-expert ICC would be higher when the Pictogram and expert groups were compared than when the Slogan/Control groups and the expert group were compared. The secondary outcome was the 2–2 between-group difference in the increase in gambling desire, with a chi-square test. Secondary analyses were performed on the subgroup without problem gambling (PGSI total score < 5). Statistics were performed with R software version 3.6.3. A flowchart is provided in Fig. 2.

### Ethics

The PictoGRRed study was approved by the Local Nîmes Hospital IRB (Approval Number 0.10.03). All experiments were performed in accordance with relevant guidelines and regulations.

## Results

### Online Delphi consensus method

The Delphi method reached a consensus on the 10 characteristics to be illustrated by pictograms in three rounds (Table 1).

In Round 1, 56 experts fulfilled the inclusion criteria and 48 (91%) rated the addictive characteristics. Two characteristics reached consensus without requiring rewording: in-game actions overly suggesting control of the outcome, and near-miss and near-miss equivalent situations. Two characteristics were excluded for low priority: avatar/customization and use of a familiar context. Eight new characteristics were added: complexity, community, cashless version available, informational asymmetry, dynamic features, facilitation of multiplying the bet, complicated unsubscription or self-exclusion, and game in the game.

In Round 2, 47 experts rated the addictive characteristics. Consensus was reached for 10 characteristics, leading to a pool of 12 characteristics, ranked in decreasing order of priority: messages associated with the game suggesting control of chance, unlimited temporal access to the game, messages promoting ease of gambling, in-game actions overly suggesting control of the outcome, fast game, near miss and near-miss equivalent situations, losses disguised as wins, high event frequency, short payout interval, in-running betting, misleading winning-related sensory feedback for a non-winning outcome, and frequent betting opportunities.

In Round 3, 52 experts rated the proposal to merge three potentially overlapping characteristics—unlimited temporal access to the game, frequent betting opportunities, and high event frequency—into “very large temporal access to the game (game available over extended hours implying that one can bet several times successively without a cooling off period, and throughout the day)”. This proposal was not retained by the experts: only 75% of the experts agreed or strongly agreed with it. Thus, the two characteristics with the lowest level of consensus were excluded, i.e., misleading winning-related sensory feedback for a non-winning outcome and frequent betting opportunities.

Table 1 presents the results of the 10 final retained characteristics. The detailed results of the Delphi process are shown in Supplementary Table 1.

Experts repeatedly reported concerns about a possible opposite effect of a pictogram “proposition/use of bonus/free spins.” They suggested that it could attract gamblers to play that specific game if they were alerted to the presence of bonuses.

### Development of the PictoGRRed pictograms and their definitions

All pictograms were presented with the following headings: “This pictogram regards a gambling product…”. All 10 characteristics were reworded to be understandable by laypeople.

### Impact of the PictoGRRed pictograms and their definitions on the assessment of the addictiveness of gambling products

We included 900 people, 300 in each group. Participant characteristics are detailed in Table 2 (no missing data, answers were required).

**Table 2:** Characteristics of the whole sample and by group (study in laypeople).

	All (*n* = 900)	Pictograms (*n* = 300)	Slogan (*n* = 300)	Control (*n* = 300)
	Mean (SD) or *N*, %	Mean (SD) or *N*, %	Mean (SD) or *N*, %	Mean (SD) or *N*, %
Age (years)	49.60 (16.60)	48.97 (16.51)	49.68 (16.57)	50.17 (16.74)
Sex (male)	430, 47.78%	137, 45.67%	142, 47.33%	151, 50.33%
Place of living (rural)	151, 16.78%	56, 18.67%	50, 16.67%	45, 15%
Employed (yes)	520, 57.78%	183, 61%	164, 54.67%	173, 57.67%
Education (undergraduate)	238, 26.44%	76, 25.33%	84, 28%	78, 26%
PGSI total score	2.33 (5.07)	2.32 (5.10)	2.60 (5.47)	2.09 (4.60)
Problem gambler (PGSI ≥ 5)	131, 14.56%	43, 14.33%	46, 15.33%	42, 14%
**Gambling practice (last 12 months)**				
None	273, 30.33%	92, 30.67%	89, 29.67%	92, 30.67%
Online lottery	241, 26.78%	80, 26.67%	82, 27.33%	79, 26.33%
Online speed lottery	175, 19.44%	53, 17.67%	68, 22.67%	54, 18%
Online scratching	73, 8.11%	21, 7%	29, 9.67%	23, 7.67%
Online horse betting	115, 12.78%	34, 11.33%	42, 14%	39, 13%
Online sport betting	64, 7.11%	22, 7.33%	19, 6.33%	23, 7.67%
Online poker	49, 5.44%	17, 5.67%	18, 6%	14, 4.67%
Online casino games	40, 4.44%	14, 4.67%	14, 4.67%	12, 4%
Offline lottery	285, 31.67%	97, 32.33%	95, 31.67%	93, 31%
Offline speed lottery	387, 43%	127, 42.33%	136, 45.33%	124, 41.33%
Offline scratching	65, 7.22%	21, 7%	26, 8.67%	18, 6%
Offline horse betting	78, 8.67%	23, 7.67%	26, 8.67%	29, 9.6%
Offline sport betting	34, 3.78%	11, 3.67%	11, 3.67%	12, 4%
Offline poker	87, 9.67%	13, 4.33%	29, 9.67%	29, 9.67%
Offline casino games	41, 4.56%	13, 4.33%	14, 4.67%	14, 4.67%

*PGSI* problem gambling severity index.

Experts and laypeople ranked the games differently: mean addictiveness of gambling products ranged from 1.09 (offline lottery) to 3.61 (online casino games) in experts, and from 2.03 (offline sport betting) to 2.56 (offline casino games) in laypeople. In the Control group, illustrating the baseline knowledge of laypeople, estimated addictiveness ranged from 1.95 (offline sport betting) to 2.44 (offline casino games). Figure 1 represents ratings from the three groups and from experts. Mean addictiveness, all games combined, was scored as 2.24 (1.05) in the Pictogram group, 2.26 (1.13) in the Slogan group, and 2.14 (1.13) the Control group. Addictiveness of the 14 gambling products ranged from 2.09 to 2.63 in the Pictogram group, from 2.11 to 2.61 in the Slogan group, and from 1.95 to 2.44 in the Control group.

**Figure 3:**
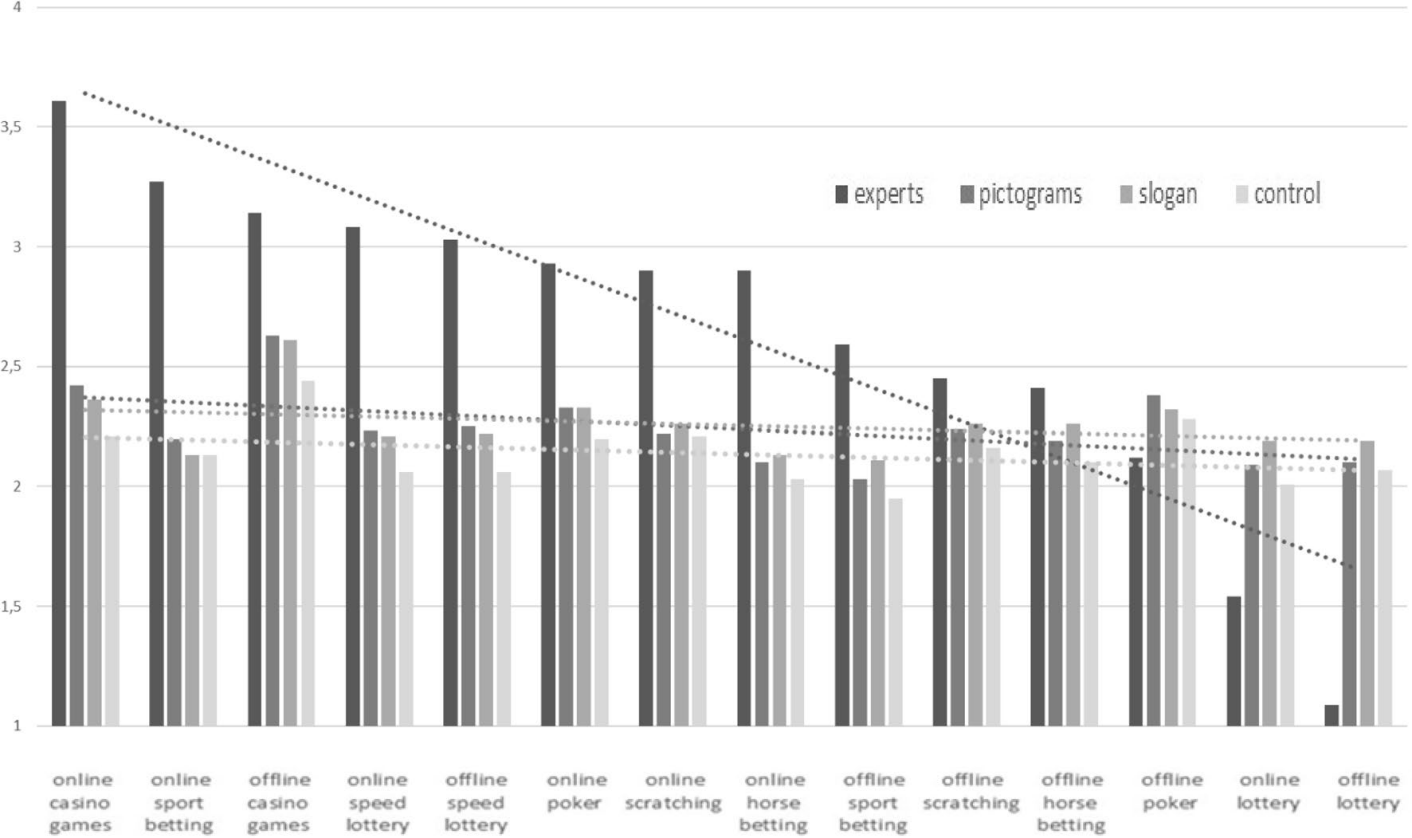
Mean addictiveness of gambling products, standard deviation and linear mean curve, as rated by the three groups (n = 900) and by French experts (n = 25) (Likert-scale 0 to 4).

Experts rated all gambling products as more addictive than the total laypeople cohort did, except for online and offline lottery and offline poker. In the Pictogram group, the ICC was approximately twice the ICC for the Slogan and Control groups (ICC = 0.28 vs. 0.14 (Slogan) and 0.14 (Control)). Thus, the pictograms approximately doubled the ability of the lay sample to identify the addictiveness of gambling products. This is illustrated by the Pictogram Group's linear mean curve, the slope of which tended to follow that of the experts' curve, in contrast to slope of the Slogan group’s curve, which remained parallel to that of the Control group (Fig. 3).

Nevertheless, the level of agreement with the experts’ rating was generally low across the three groups.

Secondary analyses of the group with no problem gambling (*n* = 769) confirmed these results: ICC = 0.29 (Pictogram-experts) vs. 0.18 (Slogan-experts) and 0.18 (Control-experts). Mean addictiveness, all games combined, was 2.23 (1.07) in the Pictogram subgroup, 2.23 (1.18) in the Slogan subgroup, and 2.09 (1.18) in the Control subgroup (analysis of variance [ANOVA] *df* = 2, *F* value = 1.07, *p* = 0.342).

Mean craving decreased in all groups (means and standard deviations): Pictogram group: -0.15 (0.40), Slogan group: -0.11 (0.34), Control group: -0.10 (0.37). The magnitude of the decrease was highest in the Pictogram group, but did not reach significance (ANOVA *df* = 2, *F* = 1.51, *p* = 0.222). Only 1.67% (*n* = 5) of the subjects in the Pictogram group declared that craving increased after seeing the pictograms and answering the baseline demographics and gambling habits questions vs. 0.67 (*n* = 2%) in the Slogan group and 2.33% (*n* = 7) in the Control group. Notably, craving decreased in 17.00% (*n* = 51) of subjects in the Pictogram group vs. 12.00% (*n* = 36) in the Slogan group and 12.67% (*n* = 38) in the Control group.

## Discussion

We conducted a multistep study to develop 10 PictoGRRed pictograms representing addictive features of gambling products, based on a Delphi consensus method involving 56 experts from 13 different countries. These pictograms illustrate the 10 main addictive structural characteristics of gambling products that laypeople should be aware of to protect themselves and reduce risky gambling in a universal or selective prevention approach. The PictoGRRed pictograms illustrate the following concepts: messages associated with the game suggesting control of chance, unlimited temporal access to the game, messages promoting ease of gambling, in-game actions overly suggesting control of the outcome, fast game, near miss and near-miss equivalent situations, losses disguised as wins, high event frequency, short payout interval, and in-running betting. Exposure to the pictograms and their definitions doubled the ability of laypeople to assess the addictiveness of gambling products compared with those who were exposed to a slogan or to nothing. Our results suggest that the slogan globally increases risk perception without distinction between gambling products and that the pictograms and their definitions increased both risk perception and discrimination of addictiveness between products compared with no exposure. Evaluation of a large sample of laypeople confirmed the safety of these pictograms, showing that they did not increase craving.

The 10 consensus concepts illustrated by the PictoGRRed pictograms are in line with those identified as important in the existing literature on gambling risks^6,11,9^. Interestingly, two concepts chosen by the experts concerned the advertising messages associated with the gambling products, either in the instructions or the environment. Both messages, which promoted illusion of control and ease of gambling, are thought to target cognitive distortions. The intensity of belief in cognitive distortions is related to the severity of gambling problems^4,29^. Prior knowledge of the nature and consequences of such messages on loss of control could have a protective effect in the general population, irrespective of advertising regulations^30^. Statistical knowledge of gambling is known to be less relevant for prevention of problem gambling than is targeting of misconceptions such as illusion of control^12,31,32^. Although the experts were instructed to select addictive factors, some factors retained in the final pool could overlap with harmful use factors and not strictly addictive factors.

Our study also showed that laypeople greatly underestimate the addictiveness of gambling products, supporting the use of the pictograms and their definition for empowerment of laypeople in protecting themselves from risky gambling. This underestimation could also be related to a propensity to underestimate personal susceptibility to addiction when exposed to gambling products. The sparse data available on the knowledge of laypeople on gambling addiction mostly focus on adolescents^33^. Laypeople showed no innate ability to identify addictiveness of gambling products when experts’ ratings were used as the reference^34^.

Online gambling combines several addictive features, incorporating unlimited temporal access, speed of play, short payout intervals, and messages often sent to gamblers. However, laypeople underestimate online gambling risks, particularly regarding online casino and online sport betting, for which the agreement was lowest between laypeople and experts. These two gambling activities are monitored by responsible gambling authorities, particularly in France, where online casino betting is illegal and online sport betting has exponentially increased in recent years^35,36^. The population could therefore have underestimated their risks because of the relative novelty of these forms of gambling. It is critical to inform the general population of these unintuitive risks for which they lack experience.

Encouragingly, the PictoGRRed pictograms and their definitions were safe to use and demonstrated a rapid, though modest, improvement in ability to assess addictive risks of gambling products after a single exposure. The pictograms could help convey these unintuitive concepts presented in the associated definition and could aid memorization by the general population, who are poorly aware of addictive structural components of gambling products. Although it could be difficult for some people to parse some of the complex concepts reduced to a picture format alone, this was not the focus of this study. Pictograms and their definitions could be included in universal and selective prevention programs that target empowerment of laypeople confronted with gambling products, including minors. The present study is a preliminary step toward developing these improved, more streamlined ways of communicating risk to the general population, and their effectiveness may depend on their implementation and support for implementation. Following the example of the PEGI pictograms, regulatory authorities could impose their use for transparency and to empower laypeople from a risk reduction and selective prevention perspective. Our approach endorsed a more balanced risk reduction approach to gambling, recognizing the addictiveness of gambling products and not delegating responsibility to potentially poorly informed gamblers. Most so-called responsible gambling interventions ask gamblers to behave responsibly, implying that they would be responsible for loss of control and addiction. This approach may be perceived as stigmatizing for people presenting a gambling disorder, and stigma is known to be very high in the general population^37^. PictoGRRed pictograms could return the responsibility to operators and regulators, helping laypeople recognize that gambling products are not harmless or ordinary commodities and can have structural addictive characteristics that drive the development and maintenance of addiction.

Our study presents some limitations. The assessment of the impact of PictoGRRed pictograms was performed in a French sample, and baseline knowledge of gambling of this cohort could be partly socio-culturally dependent. However, the use of three groups, including a non-exposed control group, considered to represent the baseline knowledge of laypeople, allowed measurement of a proxy of the progression of knowledge after exposure to a minimal prevention slogan and to the pictograms and their definitions. We collected no detailed information on how they assimilated the information provided by the pictograms and their definitions. It might be interesting in a future study to present laypeople with the pictographs and their meaning and ask them which pictograph(s) they would attach to different gambling games. Moreover, as Delphi experts were from 13 different countries, the pictograms and their definitions are highly transcultural.

Another limitation is the choice of only 10 pictograms, despite 12 concepts achieving consensus in the Delphi process. However, as these concepts are not intuitive and the population was naïve to them, 10 was considered ample to memorize and integrate. In addition, we did not evaluate efficacy in preventing future risky gambling in the general population, which remains to be tested in future larger studies. The use of pictograms from a therapeutic perspective or as an indicated prevention in people with a gambling disorder has not been studied here and could be explored in a future study in this difficult-to-reach population^38^.

## Conclusions

The 10 PictoGRRed pictograms and their definitions illustrate the main and consensual structural addictive characteristics of gambling products. These pictograms doubled the ability of laypeople to assess the addictiveness of gambling products after a single exposure in comparison with no exposure or exposure to a responsible gambling slogan, even though this ability remained low. In the highly stigmatizing context of gambling disorder, and with the increase in online gambling, the pictograms developed here represent a useful tool for a universally empowering prevention and for selective prevention on operators’ websites or gambling venues. The efficacy of such measures should now be tested, particularly on gambling behavior itself in real life.

## Supplementary Information

Supplementary Information.

## Data Availability

The datasets used and/or analyzed during the current study are available from the corresponding author on reasonable request.
