# Author Correction: Pictograms to aid laypeople in identifying the addictiveness of gambling products (PictoGRRed study)

**DOI:** 10.1038/s41598-023-30530-1

**Published:** 2023-03-01

**Authors:** Amandine Luquiens, Morgane Guillou, Julie Giustiniani, Servane Barrault, Julie Caillon, Helena Delmas, Sophia Achab, Bruno Bento, Joël Billieux, Damien Brevers, Aymeric Brody, Paul Brunault, Gaëlle Challet-Bouju, Mariano Chóliz, Luke Clark, Aurélien Cornil, Jean-Michel Costes, Gaetan Devos, Rosa Díaz, Ana Estevez, Giacomo Grassi, Anders Hakansson, Yasser Khazaal, Daniel L. King, Francisco Labrador, Hibai Lopez-Gonzalez, Philip Newall, José C. Perales, Aurélien Ribadier, Guillaume Sescousse, Stephen Sharman, Pierre Taquet, Isabelle Varescon, Cora Von Hammerstein, Thierry Bonjour, Lucia Romo, Marie Grall-Bronnec

**Affiliations:** 1 Department of Addictology, CHU Nîmes, Univ Montpellier, Nîmes, France; 2 CESP, Univ. Paris-Sud, UVSQ, INSERM, Université Paris-Saclay, Villejuif, France; 3 EA 7479 SPURBO, CHRU BREST, Université de Bretagne Occidentale, Brest and Addictologie, Brest, France; 4 CHU de Besançon, Besançon, France; 5 QualiPsy, EE 1901, Université de Tours, Tours, France; 6 Service d’Addictologie Universitaire, CSAPA-37, CHRU de Tours, Tours, France; 7 Department of Addictology and Psychiatry Nantes, Inserm U1246, CHU Nantes, Université de Nantes, Université de Tours, Nantes, France; 8 Pôle Addiction et Précarité, Centre Hospitalier Guillaume Régnier, Rennes, France; 9 WHO Collaborating Centre for Treatment and Research in Mental Health, University of Geneva, Geneva, Switzerland; 10 IAJ - Instituto de Apoio ao Jogador, Lda, Portugal; 11 Institute of Psychology, University of Lausanne, Lausanne, Switzerland; 12 Addiction Medicine, Centre for Excessive Gambling, Lausanne University Hospitals (CHUV), Lausanne, Switzerland; 13 Louvain Experimental Psychopathology (LEP), Psychological Science Research Institute, Louvain-La-Neuve, Belgium; 14 EPITA, LRE MNSHS, Paris, France; 15 Service d’Addictologie Universitaire, Équipe de Liaison et de Soins en Addictologie, CHRU de Tours, Tours, France; 16 UMR 1253, iBrain, Inserm, Université de Tours, Tours, France; 17 QualiPsy, EE, Université de Tours, 1901 Tours, France; 18 Gambling and Technological Addictions Research Unit, University of Valencia, Valencia, Spain; 19 Department of Psychology, Centre for Gambling Research at UBC, University of British Columbia, Vancouver, BC Canada; 20 Centre for Excessive Gambling, Université Catholique de Louvain, Lausanne University Hospitals (CHUV), Lausanne, Switzerland; 21 Observatoire des Jeux, Paris, France; 22 Grand Hôpital de Charleroi (GHdC), Charleroi, Belgium; 23 Psychological Science Research Institute, Université Catholique de Louvain, Louvain-la-Neuve, Belgium; 24 Scientific Research and Publication Cell (CRPS), Le Beau Vallon, Namur, Belgium; 25 Centre Hospitalier Le Domaine, ULB, Braine-L’Alleud, Belgium; 26 Service Universitaire d’Addictologie de Lyon (SUAL), CH Le Vinatier, 69500 Bron, France; 27 Child and Adolescent Psychiatry and Psychology Department, Hospital Clínic Universitari de Barcelona, Barcelona, Spain; 28 University of Deusto, Bilbao, Spain; 29 BRAIN CENTER FIRENZE, Florence, Italy; 30 Clinical Addiction Research Unit, Faculty of Medicine, Malmö Addiction Center, Lund University - Gambling Disorder Unit, Region Skåne, Sweden; 31 Addiction Medicine, Department of Psychiatry, Lausanne University Hospital and Lausanne University, Lausanne, Switzerland; 32 College of Education, Psychology, & Social Work, Flinders University, Adelaide, Australia; 33 Faculty of Psychology, Complutense University, Madrid, Spain; 34 Faculty of Information and Communication, Universitat de Barcelona, Barcelona, Spain; 35 CQUniversity, Bundaberg, Australia; 36 Department of Experimental Psychology Mind, Brain and Behavior Research Centre (CIMCYC), University of Granada, Granada, Spain; 37 Département de Psychologie, EE 1901 - Equipe Qualipsy « Qualité de vie et Santé Psychologique », Université de Tours, Tours, France; 38 Lyon Neuroscience Research Center—INSERM U1028—CNRS UMR5292, PSYR2 Team, University Lyon 1, Lyon, France; 39 National Addiction Centre, King’s College, London, UK; 40 Psychiatry and Addiction Medicine Department, CHU Lille, 59000 Lille, France; 41 Univ. Lille, ULR, 4072 Lille, France; 42 PSITEC—Psychologie: Interactions Temps Émotions Cognition, 59000 Lille, France; 43 Laboratoire de Psychopathologie et Processus de Santé, Université de Paris, 92100 Boulogne Billancourt, France; 44 APEMAC, Équipe EPSAM, Université de Lorraine, 57000 Metz, France; 45 EA 4430 Clipsyd, University Paris Nanterre, Nanterre, France

Correction to: *Scientific Reports* 10.1038/s41598-022-26963-9, published online 29 December 2022

Damien Brevers was omitted from the author list in the original version of this Article. As a result, the Author contributions section now reads:

“A.L.: Conceptualization, Validation, Visualization, funding acquisition, Investigation, Methodology, Project Administration, Supervision, Writing—Original Draft Preparation and Review & Editing; M.G.: Conceptualization, Investigation, Methodology, Project Administration, Supervision, Writing—Review & Editing; J.G.: Conceptualization, Investigation, Methodology, Project Administration, Supervision, Writing—Review & Editing; S.B.: Conceptualization, Investigation, Methodology, Project Administration, Supervision, Writing—Review & Editing; J.C.: Conceptualization, Investigation, Methodology, Project Administration, Supervision, Writing—Review & Editing; H.D.: Conceptualization, Investigation, Methodology, Project Administration, Supervision, Writing—Review & Editing; T.B.: Investigation, graphical development of the pictograms, Writing—Review & Editing; L.R.: Conceptualization, Investigation, Methodology, Project Administration, Supervision, Writing—Review & Editing; M.G.-B.: Conceptualization, Investigation, Methodology, Project Administration, Supervision, Writing—Review & Editing. S.A., B.B., J.B., D.B., A.B., P.B., G.C.-B., M.C., L.C., A.C., J.-M.C., G.D., R.D., A.E., G.G., A.H., Y.K., D.L.K., F.L., H.L., P.N., J.C.P., A.R., G.S., S.S., P.T., I.V., C.V.H. were members of the Delphi panel and contributed as well in data analysis and interpretation, Review of the manuscript & Editing. All authors read and approved the final manuscript. All members of the expert panel gave consent to have their names listed in the publication.”

Additionally, the original version of this Article contained an error in Affiliation 14, which was incorrectly given as: “Epitech, Paris, France”. The correct affiliation is: “EPITA, LRE MNSHS, Paris, France”.

The original Article has been corrected.

