# Supplementary material for: Pictograms to aid laypeople in identifying the addictiveness of gambling products (PictoGRRed study)
Source: Sci Rep. 2022 Dec 29;12:22510. doi: 10.1038/s41598-022-26963-9 (PMC9800380; doi:10.1038/s41598-022-26963-9)
Supplement: Supplementary file 1 — Supplementary Information. [file 41598_2022_26963_MOESM1_ESM.docx]

Supplementary Table 1: Initially proposed and elicited addictive charactseristics of gambling products and related messages, results of the Delphi process, and final 10 pictograms and their reworded definitions for laypeople

| Addictive structural characteristics of gambling products and related messages | Results of priority rating in round 1 | | | | | | | Rewording for loop 2 following comments of experts in loop 1 | Results of priority rating in round 2 | | | | | Pictogram | | Definition reworded for laypeople:  “This pictogram regards a gambling game .…” |
| --- | --- | --- | --- | --- | --- | --- | --- | --- | --- | --- | --- | --- | --- | --- | --- | --- |
|  | Not a priority | Low priority | | Medium priority | | High Priority | Essential |  | Not a priority | Low priority | Medium priority | High Priority | Essential |  |  |  |
| *Initially proposed characteristics* | | | | | | | | | | | | | |  | |  |
| **Frequent betting opportunities** (possibility to bet several times successively so that the gambler keeps gambling during a certain period of time) | 0 (0.0%) | 2 (4.8%) | | 9 (21.4%) | | 18 (42.9%) | 13 (31.0%) | { One has never long to wait until the next opportunity to bet/stake | 0 (0.0%) | 0 (0.0%) | 7 (14.9%) | 36 (76.6%) | 4 (8.5%) |  | |  |
| **High event frequency** (virtually unlimited opportunity to play) | 0 (0.0%) | 1 (2.4%) | | 9 (21.4%) | | 18 (42.9%) | 14 (33.3%) | { Possibility to bet several times successively so that the gambler keeps gambling during a certain period of time, virtually unlimited opportunity to play | 0 (0.0%) | 0 (0.0%) | 8 (17.0%) | 28 (59.6%) | 11 (23.4%) | **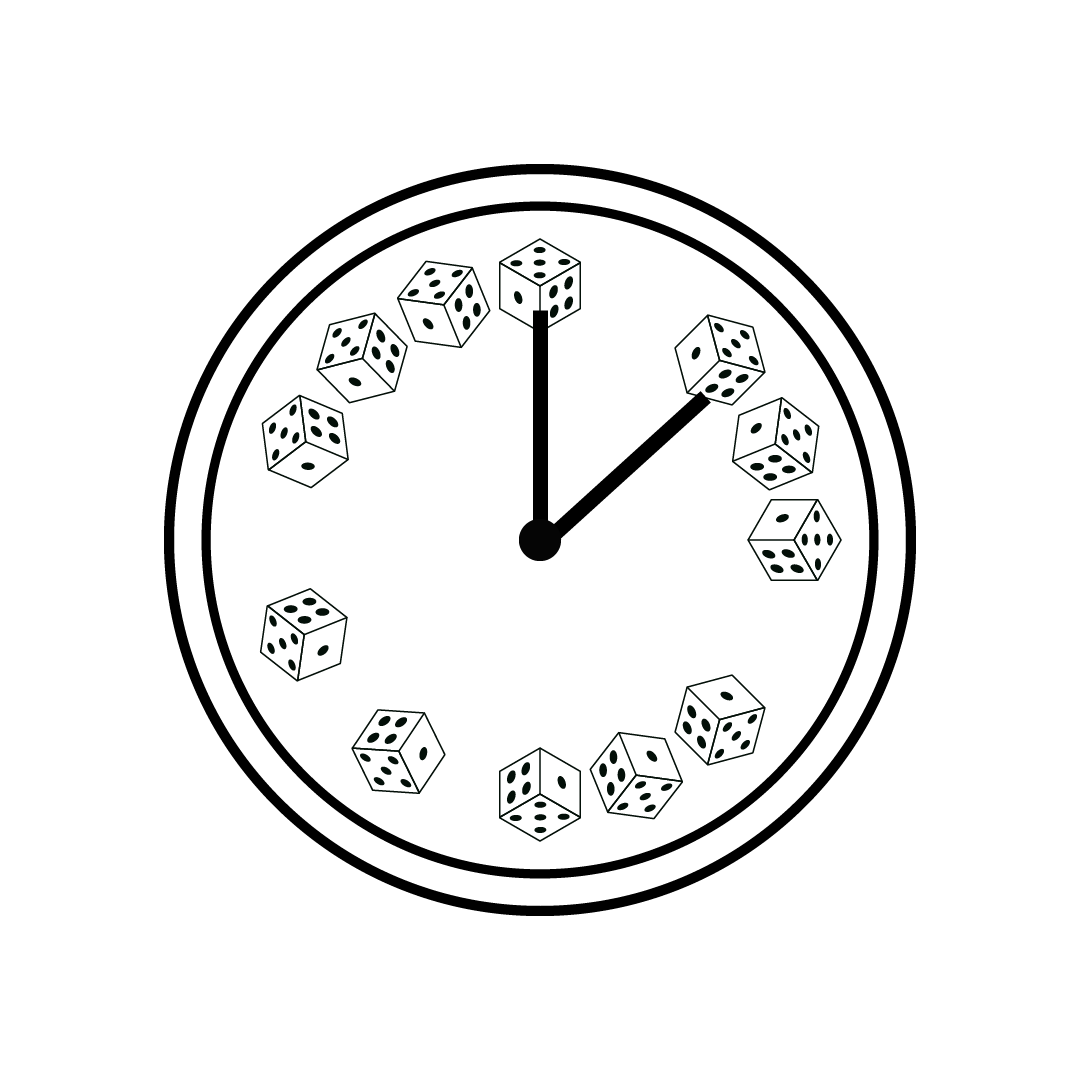** | | "... where it is possible to bet several times in a row so that the gambler can continue to play practically without a break." |
| **Behavioral learning and rapid feedback** (the player has to learn a certain behavior and rapidly receives feedback on his or her performance) | 4 (9.5%) | 10 (23.8%) | | 14 (3.3%) | | 9 (21.4%) | 5 (11.9%) |  | 3 (6.4%) | 11 (23.4%) | 20 (42.6%) | 11 (23.4%) | 2 (4.3 %) |  | |  |
| **Progressive gains linked to immersion/in-game progress** (the player has to go through a number of successive challenges to have the opportunity of gains) | 3 (7.1%) | 7 (16.7%) | | 17 (40.5%) | | 14 (33.3%) | 1 (2.4%) |  | 1 (2.1%) | 15 (31.9%) | 21 (44.7%) | 10 (21.3%) | 0 (0.0%) |  | |  |
| **Fast game** (high event frequency and limited scope for decision making) | 0 (0.0%) | 0 (0.0%) | | 14 (33.3%) | | 14 (33.3%) | 14 (33.3%) |  | 0 (0.0%) | 0 (0.0%) | 5 (10.6%) | 25 (53.2%) | 17 (36.2%) | **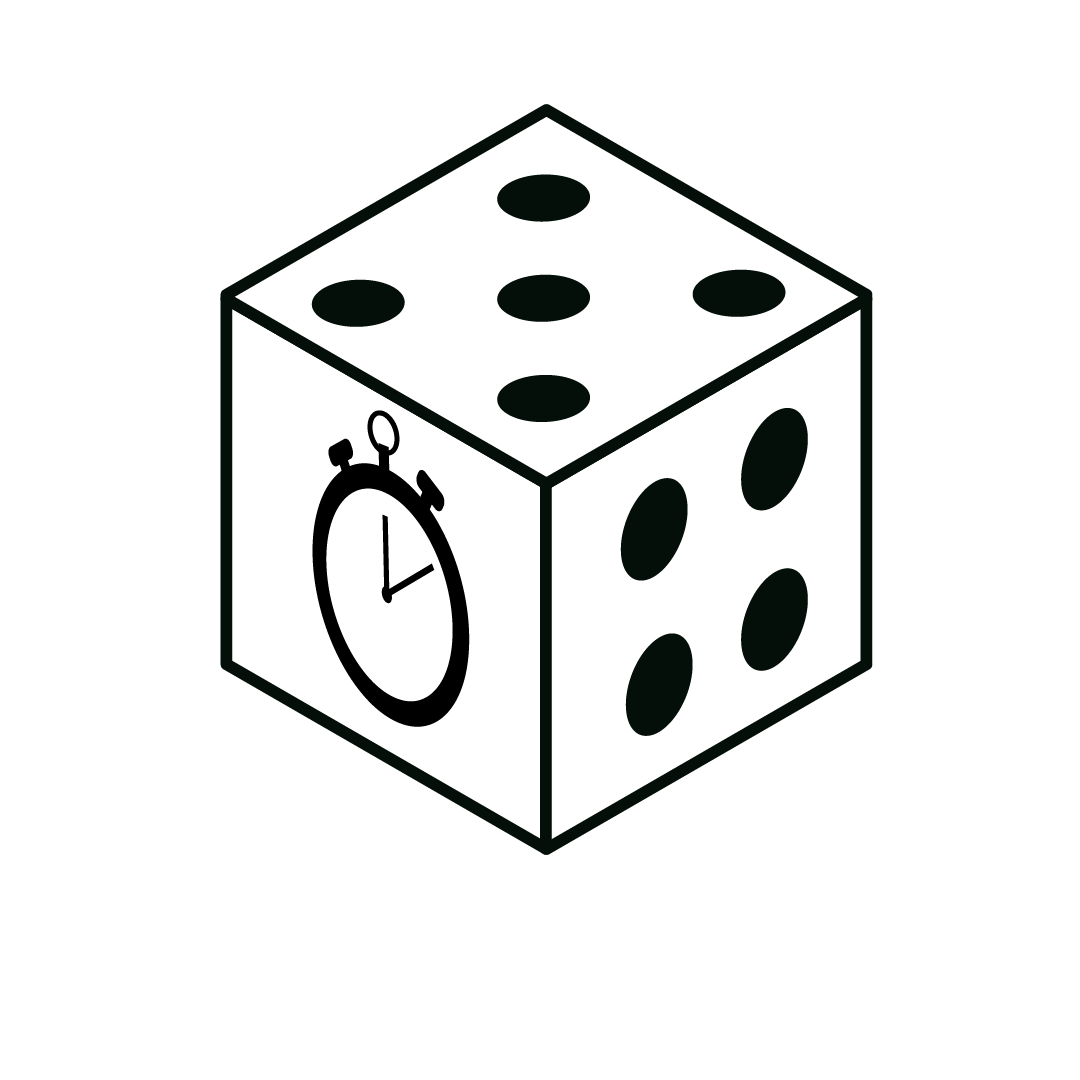** | | “...where it is possible to wager frequently but with a limited time to think before each wager.” |
| **In-game actions overly suggesting control of the outcome** (in-game actions suggesting control of chance, whereas they do not or only poorly influence the outcome) | 0 (0.0%) | 0 (0.0%) | | 8 (19.0%) | | 18 (42.9%) | 16 (38.1%) |  |  |  |  |  |  | **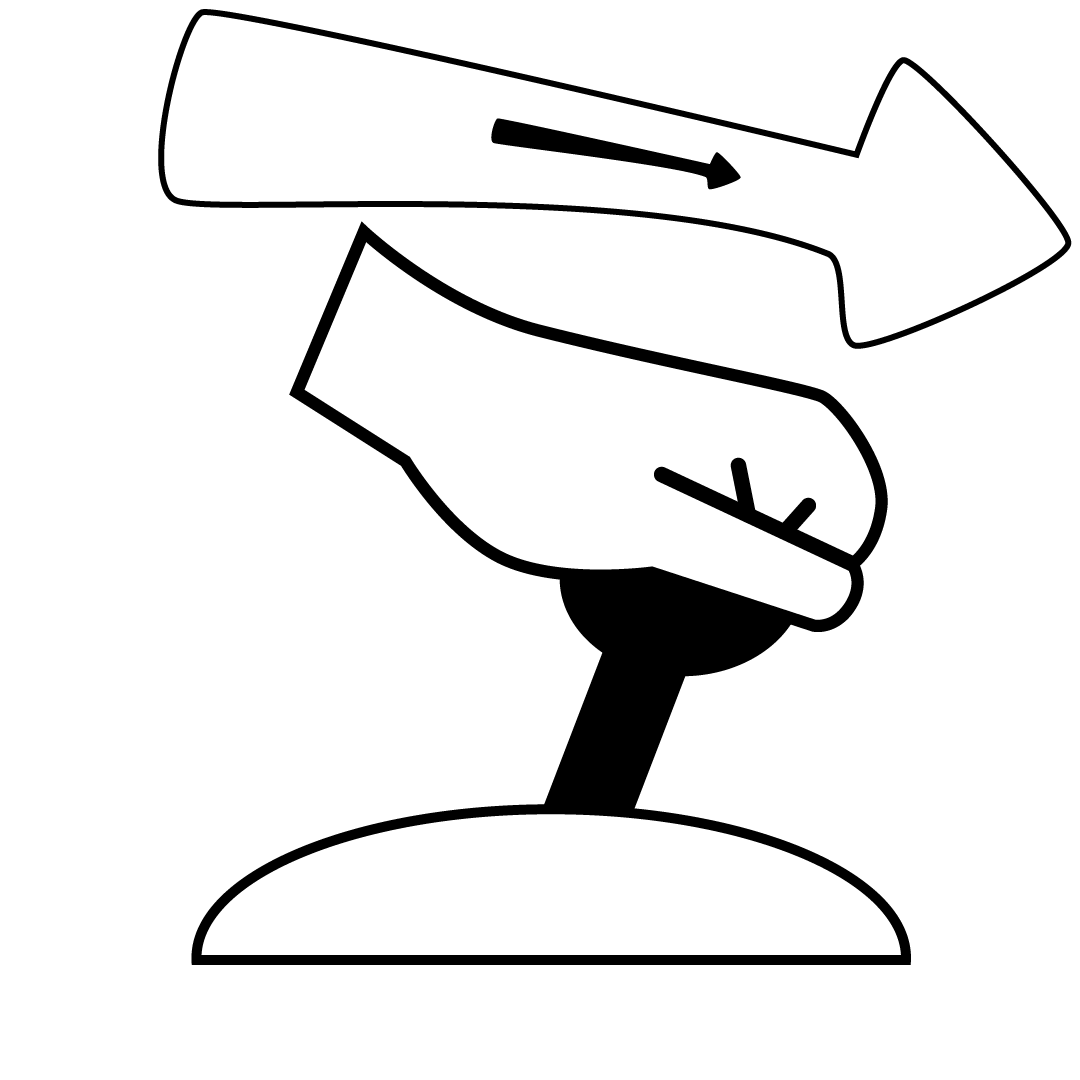** | | "...with actions that give the player the feeling that he or she can control the outcome (win/loss) when this is not the case." |
| **Autoplay** (lack of (physical) interaction, such as even pressing a button) | 1 (2.4%) | 10 (23.8%) | | 19 (45.2%) | | 9 (21.4%) | 3 (7.1%) |  | 1 (2.1%) | 12 (25.5%) | 30 (63.8) | 4 (8.5%) | 0 (0.0%) |  | |  |
| **In-running betting** (e.g., live betting) | 0 (0.0%) | 3 (7.1%) | | 11 (26.2%) | | 17 (40.5%) | 11 (26.2%) |  | 0 (0.0%) | 1 (2.1%) | 8 (17.0%) | 32 (68.1%) | 6 (12.8%) | **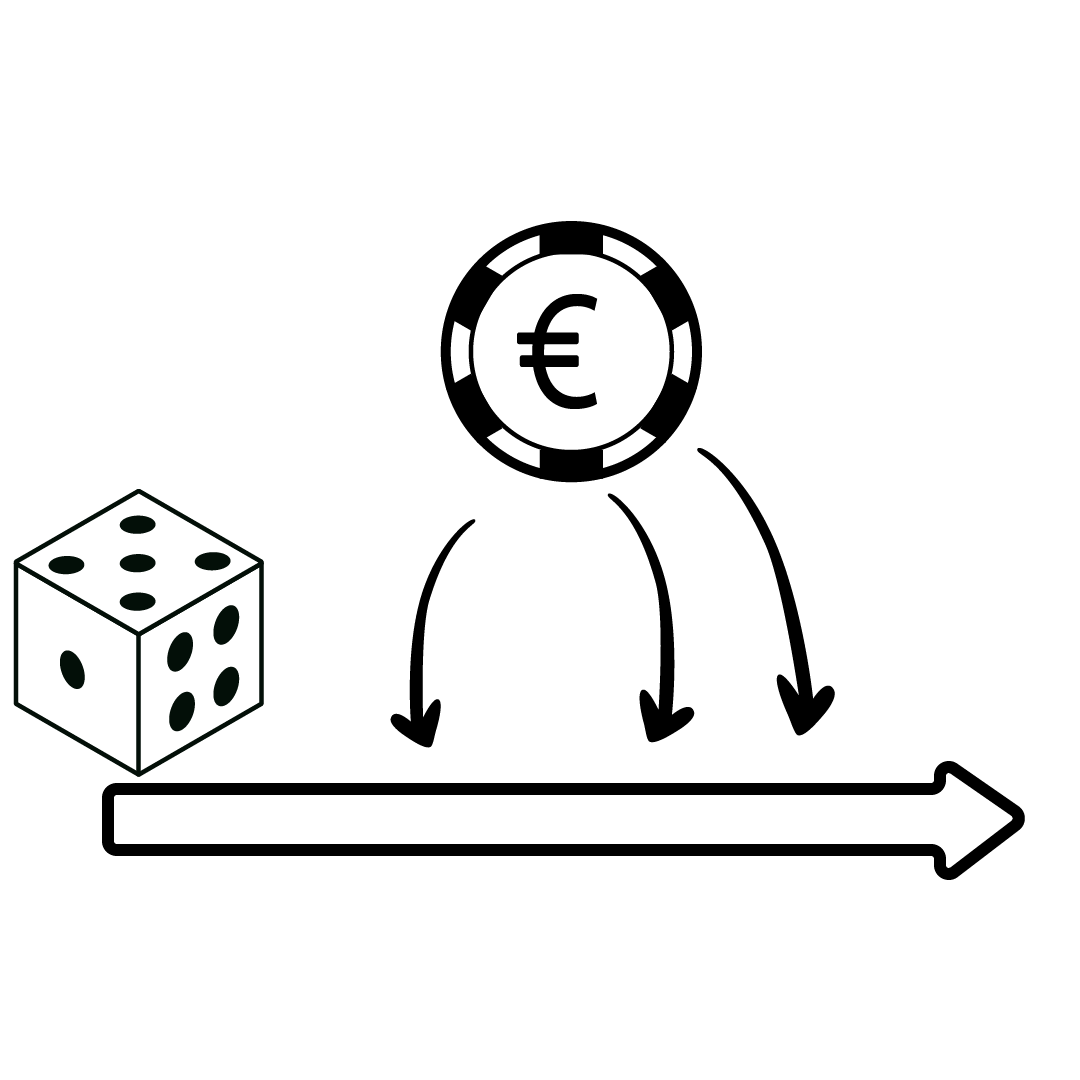** | | “"...where the gambler plays at the same time as the action is in progress (e.g., live betting, sports betting during a match)." |
| **Short payout interval** (includes rapid provision of money and automatically adding money win to credit) | 1 (2.4%) | 1 (2.4%) | | 10 (23.8%) | | 17 (40.5%) | 13 (31.0%) |  | 0 (0.0%) | 1 (2.1%) | 5 (10.6%) | 31 (66.0%) | 10 (21.3%) | 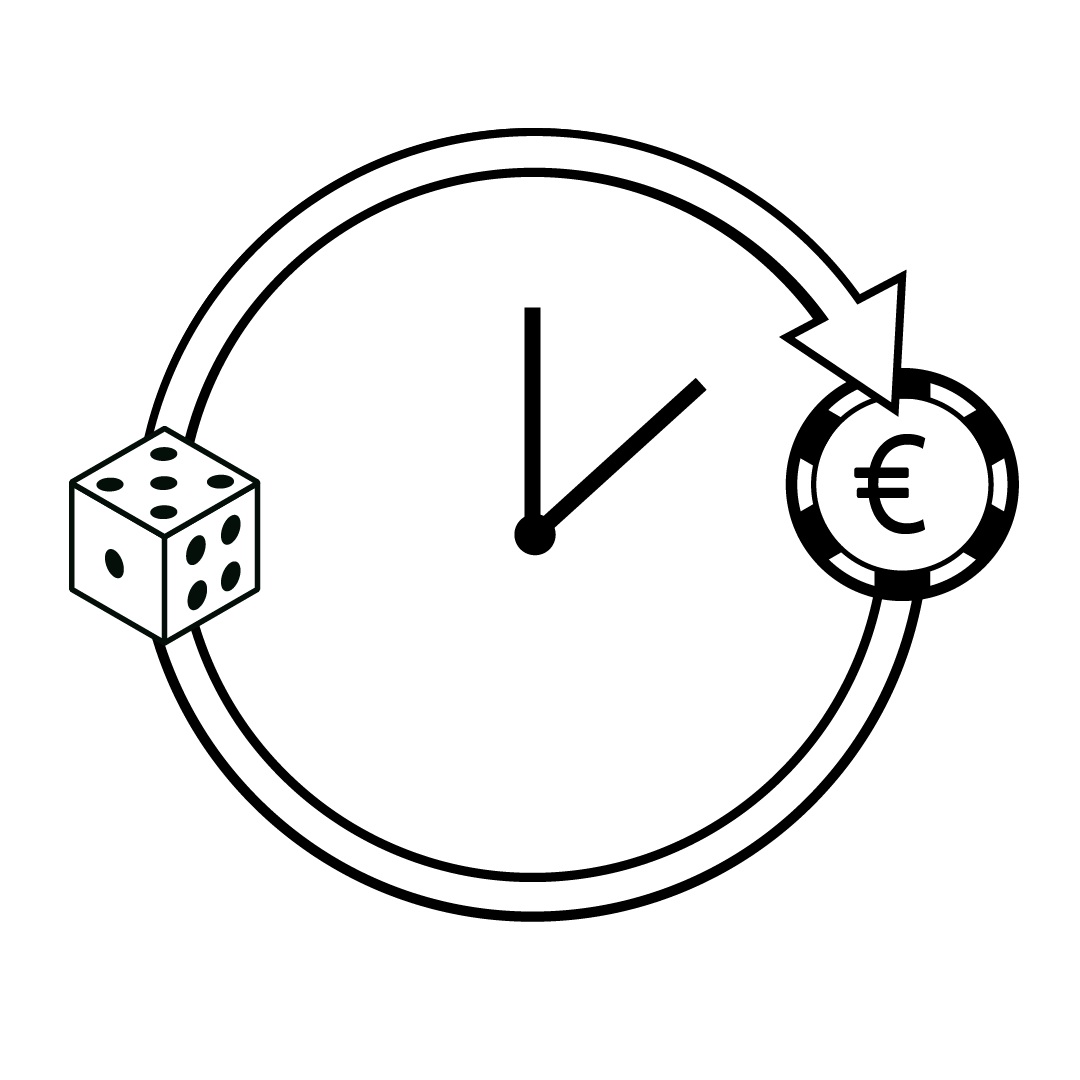 | | “...with a short payout period: the money won is very quickly paid into the gambler's account, inviting the gambler to keep on gambling.” |
| **Rapid provision of monetary** rewards (the gambler receives winnings rapidly after the outcome) | 2 (4.8%) | 5 (11.9%) | | 11 (26.2%) | | 18 (42.9%) | 6 (14.3%) |  | 0 (0.0%) | 2 (4.3%) | 11 (23.4%) | 28 (59.6%) | 6 (12.8%) |  | |  |
| Au**tomatically adding monetary rewards to total** (winnings appear directly in a total amount and not for a particular bet) | 1 (2.4%) | 10 (23.8%) | | 11 (26.2%) | | 16 (38.1%) | 4 (9.5%) |  | 1 (2.1%) | 4 (8.5%) | 15 (31.9%) | 26 (55.3%) | 1 (2.1%) |  | |  |
| **High maximum jackpot** (large amount of money announced as the maximum jackpot) | 1 (2.4%) | 11 (26.2%) | | 10 (23.8%) | | 12 (28.6%) | 8 (19.0%) |  | 0 (0.0%) | 14 (29.8%) | 15 (31.9%) | 13 (27.7%) | 5 (10.6%) |  | |  |
| **Changing maximu**m **jackpot** (the maximum jackpot is not fixed and changes over time) | 2 (4.8%) | 16 (38.1%) | | 9 (21.4%) | | 12 (28.6%) | 3 (7.1%) |  | 1 (2.1%) | 29 (61.7%) | 11 (23.4%) | 4 (8.5%) | 2 (4.3%) |  | |  |
| **Moderate volatility** (unpredictability of reward but not very low reward frequency) | 2 (4.8%) | 14 (33.3%) | | 16 (38.1%) | | 17 (16.7%) | 3 (7.1%) | Volatility (irregular interval and randomization of prizes, which acts as an intermittent reinforcement) | 0 (0.0%) | 11 (23.4%) | 29 (61.7%) | 4 (8.5%) | 3 (6.4%) |  | |  |
| **Early win** (frequent wins at the beginning of a gambling session) | 2 (4.8%) | 4 (9.5%) | | 13 (31.0%) | | 17 (16.7%) | 8 (19.0%) |  | 3 (6.4%) | 4 (8.5%) | 8 (17.0%) | 28 (59.6%) | 4 (8.5%) |  | |  |
| **Losses disguised** as wins (gain inferior to the amount staked) | 0 (0.0%) | 4 (9.5%) | | 7 (16.7%) | | 16 (38.1%) | 15 (35.7%) |  | 0 (0.0%) | 0 (0.0%) | 5 (10.6%) | 29 (61.7%) | 13 (27.7%) | **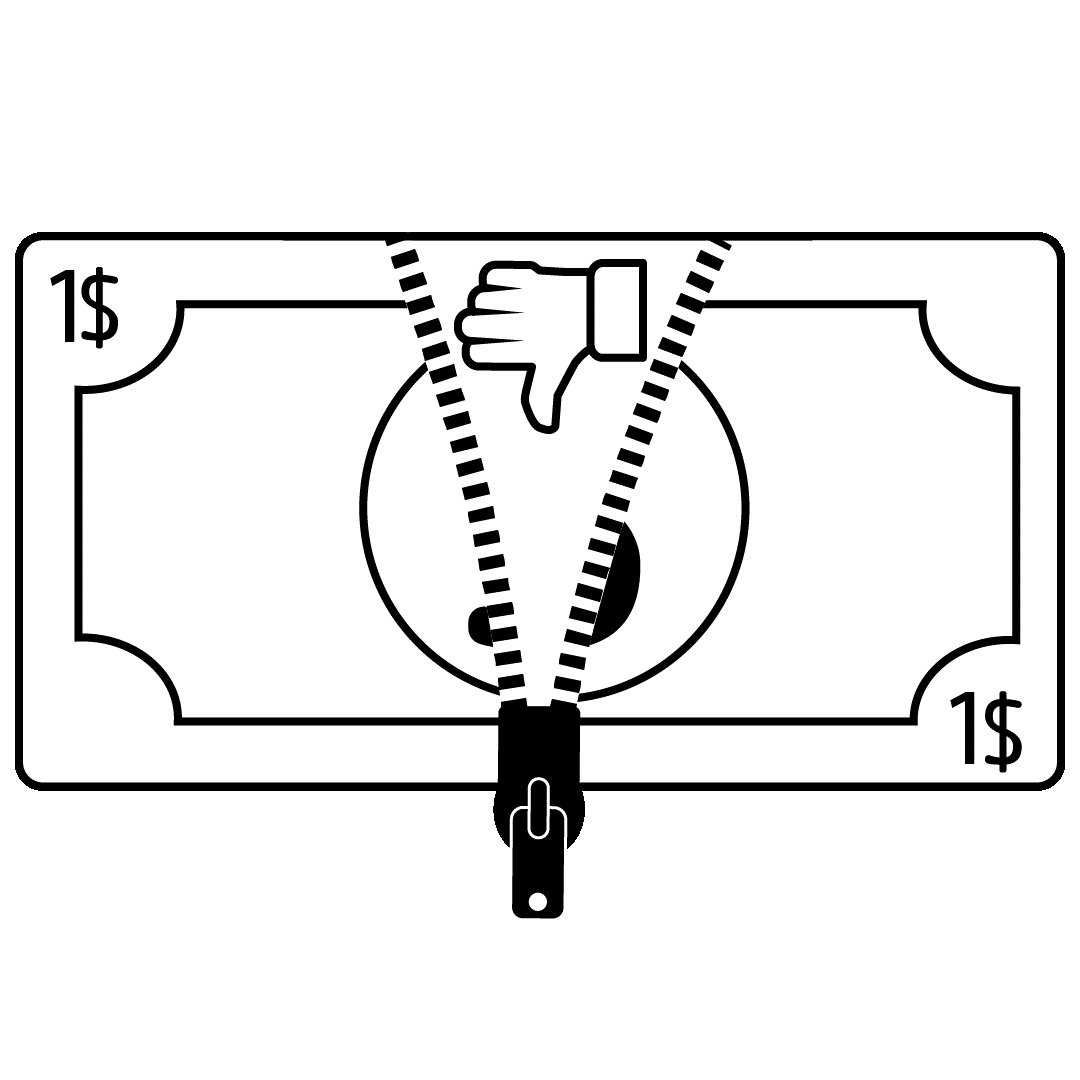** | | “...where losses are disguised as gains. For example, you buy a gambling game for 3 euros. You scratch and the gambling game indicates that you have won 1 euro, when in reality you have lost 2 euros." |
| **Misleading winning-related sensory** **stimulus for a non-winning outcome** (e.g., sounds, colors usually appearing while winning, presented for a near miss) | 0 (0.0%) | 1 (2.4%) | | 14 (33.3%) | | 18 (42.9%) | 9 (21.4%) |  | 0 (0.0%) | 2 (4.3%) | 7 (14.9%) | 32 (68.1%) | 6 (12.8%) |  | |  |
| **Near miss and equivalent of near miss** situations (outcome that suggests an outcome close to a winning situation, whereas outcome is binary: win or lose) | 0 (0.0%) | 0 (0.0%) | | 8 (19.0%) | | 20 (47.6%) | 14 (33.3%) |  |  |  |  |  |  | **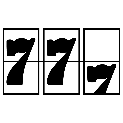** | | “...in which the outcome suggests to the gambler that he/she has ‘almost’ won, while the outcome is binary (win or lose) and he/she has lost." |
| **Possibility of high stake** (various stakes possible up to high stakes) | 0 (0.0%) | 3 (7.1%) | | 19 (45.2%) | | 15 (35.7%) | 5 (11.9%) |  | 0 (0.0%) | 5 (10.6%) | 28 (59.6%) | 14 (29.8%) | 0 (0.0%) |  | |  |
| **Extreme return-to-player rate (very low or very high)** (very high or very low value of prizes redistributed to players of the same game as a proportion of the total amount wagered on that game over the long term) | 3 (7.1%) | 5 (11.9%) | | 18 (42.9%) | | 14 (33.3%) | 2 (4.8%) | { Very high or very low value of prizes redistributed to players of the same game as a proportion of the total amount wagered on that game over the long term | 0 (0.0%) | 9 (19.1%) | 28 (59.6%) | 7 (14.9%) | 3 (6.4%) |  | |  |
| **Non-cash payment instruments** (e.g., token, bank transfer...) | 3 (7.1%) | 10 (23.8%) | | 12 (28.6%) | | 10 (23.8%) | 7 (16.7%) |  | 0 (0.0%) | 12 (25.5%) | 19 (40.4%) | 16 (34.0%) | 0 (0.0%) |  | |  |
| **No monitoring of** d**uration of play or obvious clock** | 0 (0.0%) | 5 (11.9%) | | 19 (45.2%) | | 10 (23.8%) | 8 (19.0%) |  | 0 (0.0%) | 5 (10.6%) | 25 (53.2%) | 17 (36.2%) | 0 (0.0%) |  | |  |
| **Proposition/use of bonus/free spins** (possibility to play with the operator's money) | 1 (2.4%) | 5 (11.9%) | | 13 (31.0%) | | 17 (40.5%) | 6 (14.3%) |  | 1 (2.1%) | 7 (14.9%) | 13 (27.7%) | 24 (51.1%) | 2 (4.3%) |  | |  |
| **Messages associated with the game suggesting control of chance (advertisement, instruction**) (e.g., advertisement claiming that you have particular ability over the others to win) | 1 (2.4%) | 1 (2.4%) | | 9 (21.4%) | | 12 (28.6%) | 19 (45.2%) |  | 0 (0.0%) | 0 (0.0%) | 4 (8.5%) | 16 (34.0%) | 27 (57.4%) | **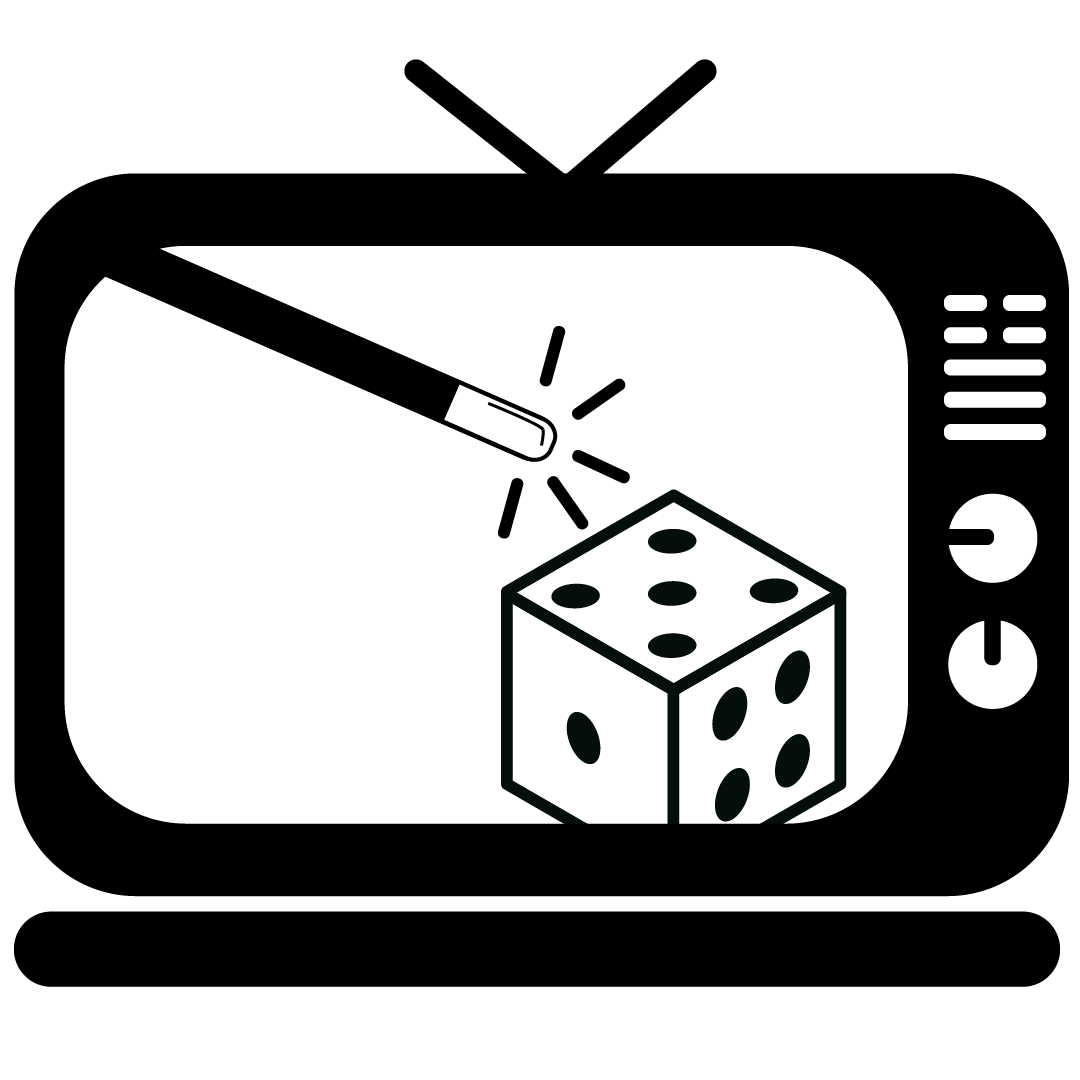** | | “"...in which the advertising messages or instructions suggest that the gambler has control over chance (e.g., the advertisement claims that the gambler has a greater ability to win than others).” |
| **Announcement of other gamblers’ gains** (e.g., big winner poster) | 2 (4.8%) | 7 (16.7%) | | 16 (38.1%) | | 11 (26.2%) | 6 (14.3%) |  | 2 (4.3%) | 8 (17.0%) | 23 (48.9%) | 12 (25.5%) | 2 (4.3%) |  | |  |
| **Customization, avatar** (tools to personalize the game) | 8 (19.0%) | 17 (40.5%) | | 13 (31.0%) | | 3 (7.1%) | 1 (2.4%) |  |  |  |  |  |  |  | |  |
| **Design using familiar context** (theme referring to familiar activity for most people, hero, movie...) | 5 (11.9%) | 16 (38.1%) | | 15 (35.7%) | | 4 (9.5%) | 2 (4.8%) |  |  |  |  |  |  |  | |  |
| **Design or advertisem**ent **using a celebrity as a spokesperson/a gambler** (e.g., TV advertisement showing a movie star playing on a poker website) | 3 (7.1%) | 14 (33.3%) | | 8 (19.0%) | | 11 (26.2%) | 6 (14.3%) |  | 6 (12.8%) | 16 (34.0%) | 12 (25.5%) | 11 (23.4%) | 2 (4.3%) |  | |  |
| **Stressful environment** (sensory overstimulation) | 2 (4.8%) | 10 (23.8%) | | 15 (35.7%) | | 10 (23.8%) | 5 (11.9%) | Stressful environment (related to the game design or venue) (e.g., sensory overstimulation, high levels of audiovisual sensory feedback) | 3 (6.4%) | 13 (27.7%) | 23 (48.9%) | 8 (17.0%) | 0 (0.0%) |  | |  |
| **Possibility of multi-playing** (multi-tabling, multiline) | 1 (2.4%) | 4 (9.5%) | | 14 (33.3%) | | 13 (31.0%) | 10 (23.8%) |  | 0 (0.0%) | 4 (8.5%) | 21 (44.7%) | 21 (44.7%) | 1 (2.1%) |  | |  |
| **Unlimited temporal access** **to the game** (24/7 venue, online…) | 0 (0.0%) | 2 (4.8%) | | 8 (19.0%) | | 13 (31.0%) | 19 (45.2%) |  | 0 (0.0%) | 0 (0.0%) | 5 (10.6%) | 16 (34.0%) | 26 (55.3%) | 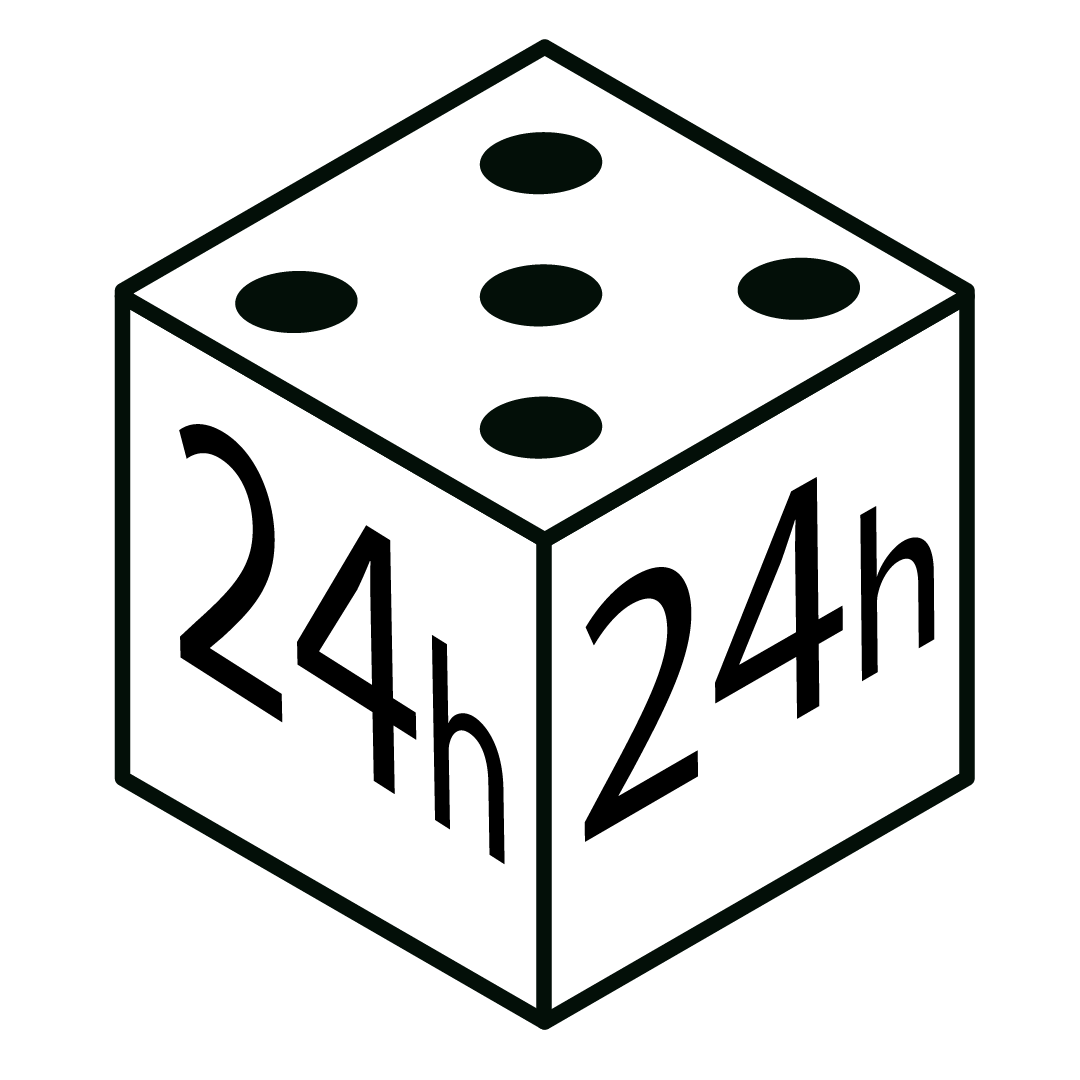 | | “  "... with an access unlimited in time (24/7, online”)." |
| **Messages promoting ease of gambling** (including bonus and rapid cash-out messages) | 1 (2.0%) | 2 (3.9%) | | 12 (23.5%) | | 16 (31.4%) | 20 (39.2%) |  | 0 (0.0%) | 3 (6.4%) | 5 (10.6%) | 17 (36.2%) | 22 (46.8%) | **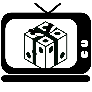**“  "... for which advertising messages or instructions encourage gambling (e.g., ‘safe first bet,’ bonus, rapid cash-out”)." |  |  |
| **Missing information** **on bet characteristics** (e.g., gross stake amount not shown) | 0 (0.0%) | 6 (14.3%) | | 14 (33.3%) | | 13 (31.0%) | 9 (21.4%) | { e.g., gross stake amount not shown. It could be that stakes do not appear directly but are presented minus previous winnings | 0 (0.0%) | 5 (10.6%) | 20 (42.6%) | 16 (34.0%) | 6 (12.8%) |  | |  |
| *New characteristics elicited by the*  *experts in phase 1* | | | | | | | | | | | | | |  | |  |
| **Complexity** (complexity of the game, which can lead to unrealistic expectations from the gamblers) | / | / | | / | | / | / |  | 2 (4.3%) | 12 (25.5%) | 22 (46.8%) | 9 (19.1%) | 2 (4.3%) |  | |  |
| **Community** (e.g., presence of a chat between gamblers, which could lead to a peer-related incentive effect) | / | / | | / | | / | / |  | 3 (6.4%) | 15 (31.9%) | 20 (42.6%) | 9 (19.1%) | 0 (0.0%) |  | |  |
| **Cashless version available**  (possibility to gamble for free in cashless/demo/practice versions of the same game) | / | / | | / | | / | / |  | 1 (2.1%) | 12 (25.5%) | 19 (40.4%) | 12 (25.5%) | 3 (6.4%) |  | |  |
| **Informational asymmetry**  (information on gamblers' behavior is processed and/or exploited by vendors) | / | / | | / | | / | / |  | 4 (8.5%) | 6 (12.8%) | 14 (29.8%) | 16 (34.0%) | 7 (14.9%) |  | |  |
| **Dynamic features** (game features adapt in response to gamblers' behavior) | / | / | | / | | / | / |  | 3 (6.4%) | 8 (17.0%) | 9 (19.1%) | 22 (46.8%) | 5 (10.6%) |  | |  |
| **Facilitation of multiplying the bet** (e.g., proposition to multiply the bet in the same round x2) | / | / | | / | | / | / |  | 1 (2.1%) | 6 (12.8%) | 15 (31.9%) | 21 (44.7%) | 4 (8.5%) |  | |  |
| **Uneasy unsubscription or self-exclusion** (easy reversal, process not available or complex to complete) | / | / | | / | | / | / |  | 0 (0.0%) | 9 (19.1%) | 8 (17.0%) | 20 (42.6%) | 10 (21.3%) |  | |  |
| **Game in the game** (e.g., random jackpot or gain added to the game, promising a multiplication of gain) | / | / | / | | / | | / |  | 0 (0.0%) | 10 (21.7%) | 17 (37.0%) | 16 (34.8%) | 3 (6.5%) |  | |  |
| *..….. Concepts that reached consensus for retention since the first round*  *……... Concepts that reached consensus for exclusion at 1^st^ or 2^nd^ round*  *…….. Factors not reworded between the 2 rounds or elicited in phase 1*  Only the explanation was reworded | | | | | | | | | | | | | |  | |  |
